# Off-label use of large diameter Concerto fibered coils through a 0.017 inch microcatheter for transvenous embolization of indirect carotid-cavernous fistulas: two case reports

**DOI:** 10.1186/s13256-024-04344-2

**Published:** 2024-02-05

**Authors:** Justin T. Hsieh, Ghim Song Chia, Chen Pong Wong, Winston Eng Hoe Lim, David W. Wen

**Affiliations:** Department of Diagnostic Radiology, Singapore General Hospital, Singapore, Singapore

**Keywords:** Carotid-cavernous fistulas, Transvenous embolization, Fibered coils, Neurointervention

## Abstract

**Background:**

A carotid-cavernous fistula is an abnormal communication between the arteries and veins within the cavernous sinus. While conservative management may be prudent in low risk cases, many patients require intervention and endovascular embolization has evolved as the preferred method of treatment. Embolization can be performed via either the transarterial or transvenous approach. One major challenge of the transvenous approach is the complex and variable compartmentation of the cavernous sinus, which often requires the use of low profile microcatheters to navigate and reach the fistulous point. Fibered coils are also preferred when performing transvenous embolization of carotid-cavernous fistula, as they are of higher thrombogenicity and allow for faster occlusion of the fistula. However, most low profile (0.017-inch) microcatheters are not able to deploy fibered coils based on the manufacturer’s instructions.

**Case presentation:**

We present two successful cases of off-label use of Medtronic Concerto fibered coils via a 0.017-inch microcatheter during transvenous embolization of carotid-cavernous fistula in a 60-year-old and an 80-year-old Chinese female, respectively.

**Conclusion:**

Our case series highlight the possibility of deploying large diameter (up to 10 mm) Concerto fibered coils through a low profile (0.017-inch) microcatheter in an off-label manner for transvenous embolization of indirect carotid-cavernous fistula.

## Introduction

A carotid-cavernous fistula (CCF) is an abnormal arteriovenous communication between the cavernous portion of the internal carotid artery (ICA) and the cavernous sinus. CCF is largely divided into direct and indirect subtypes: direct CCF is characterized by direct connection between the ICA and the cavernous sinus, while indirect (or dural) CCF consists of connection between arterial branches of either internal or external carotid arteries and the cavernous sinus. In patients whose vision are threatened or suffer from symptoms that affect quality of life, treatment is often recommended. Endovascular embolization has been considered first line [1, 2] and can be performed via the transarterial or transvenous approaches [3].

In the case of indirect CCFs, the transvenous route is often the safer and more efficacious approach for embolization [4]. However, due to the complex and variable compartmentation of the cavernous sinus, navigating to the fistulous point often requires a low profile microcatheter such as one with a 0.017-inch inner diameter. The down side of low profile microcatheters is that they are not compatible with most detachable fibered coils. We prefer using large diameter fibered coils for transvenous embolization of indirect CCF due to superior thrombogenicity. This allows for faster occlusion of the fistula with less coils.

In our practice, although not recommended based on the manufacturer’s instructions for use (IFU), we have found that it is possible to deploy large diameter Concerto nylon fibered coils (Medtronic, Irvine, California) through a 0.017-inch microcatheter [8]. We present two cases to illustrate our technique and outcomes.

### Case presentation 1

A 60-year-old Chinese female presented with a 3-day history of nontraumatic diplopia and mild left eye ptosis. Clinical examination revealed a left third cranial nerve palsy. Urgent computed tomography (CT) angiogram demonstrated arterial enhancement in the left cavernous sinus. Catheter angiography confirmed left indirect CCF supplied by the inferolateral trunk (ILT) of the left internal carotid artery (ICA). The fistula drained from left cavernous sinus into the right cavernous sinus and subsequently into the right inferior petrosal sinus (IPS) then into the right internal jugular vein (IJV).

Embolization was performed via a right brachial vein access. A 6F guide-catheter was positioned in the right IJV. Through the guide catheter, a Headway 17 (MicroVention) microcatheter was navigated into the left cavernous sinus fistulous point. Multiple Concerto nylon fibered coils were deployed (sizes range from 5 to 7 mm in diameter) until there was significant reduction in arterial flow through the fistula. No complication occurred during the procedure.

Patient recovered well with complete resolution of her diplopia and left eye ptosis at her 6-month follow-up appointment. Magnetic resonance imaging (MRI) performed 12 months after the embolization did not show any residual CCF.

### Case presentation 2

**Fig. 1:**
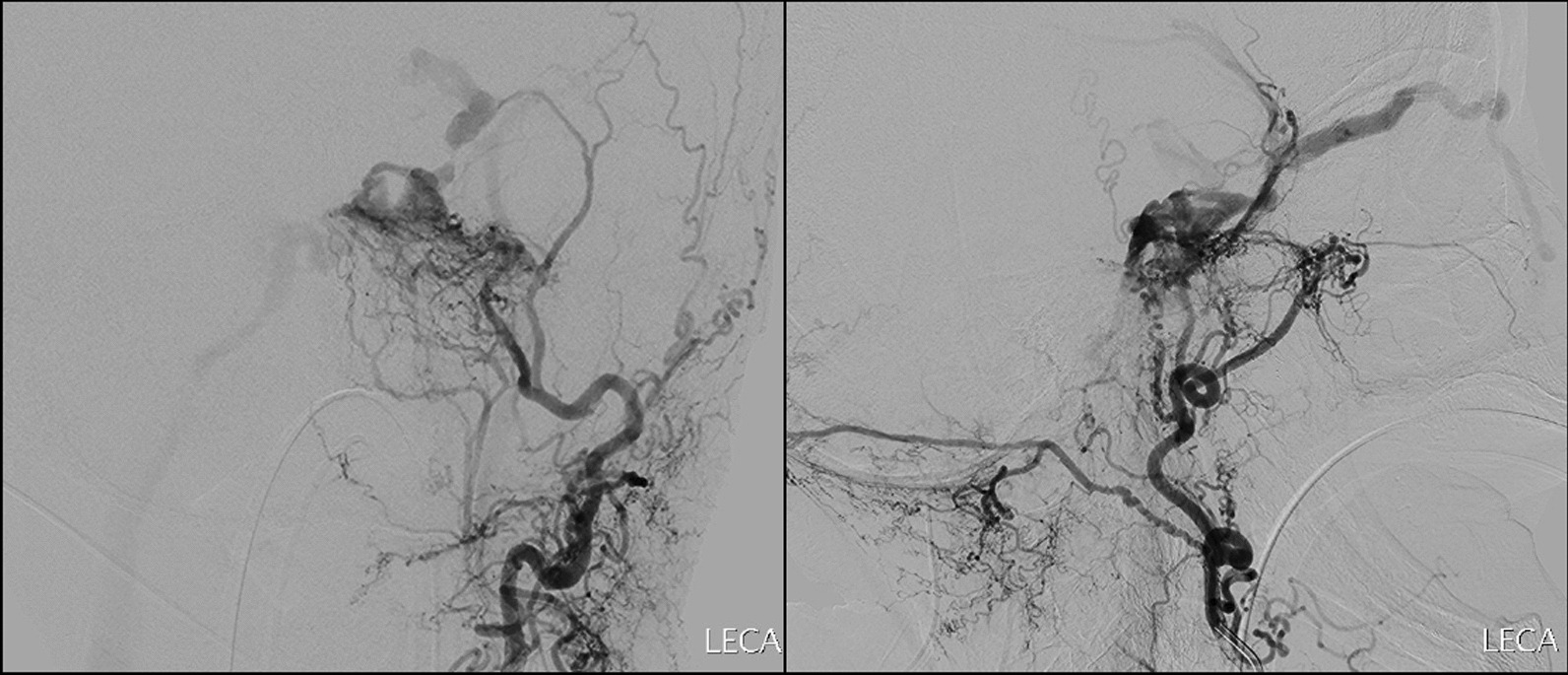
Angiogram of the left external carotid artery in anterior-posterior (left) and lateral (right) views demonstrate indirect carotid-cavernous fistula of case 2. Note venous drainage into the right inferior petrosal sinus and right superior ophthalmic vein

An 80-year-old Chinese female presented with a 1-month history of nontraumatic right eye esotropia. Clinical examination revealed bilateral sixth cranial nerve palsies, worse on the right. MRI demonstrated enlarged bilateral superior ophthalmic veins and prominent flow voids in the bilateral cavernous sinuses. Catheter angiography confirmed bilateral indirect CCF supplied by bilateral external carotid artery (ECA) branches, including the ascending pharyngeal, middle meningeal and internal maxillary arteries. Retrograde venous drainage into the bilateral superior ophthalmic veins and antegrade venous drainage into the right IPS were noted (Fig. 1). Cortical venous reflux into the left superficial middle cerebral vein was also seen.

**Fig. 2:**
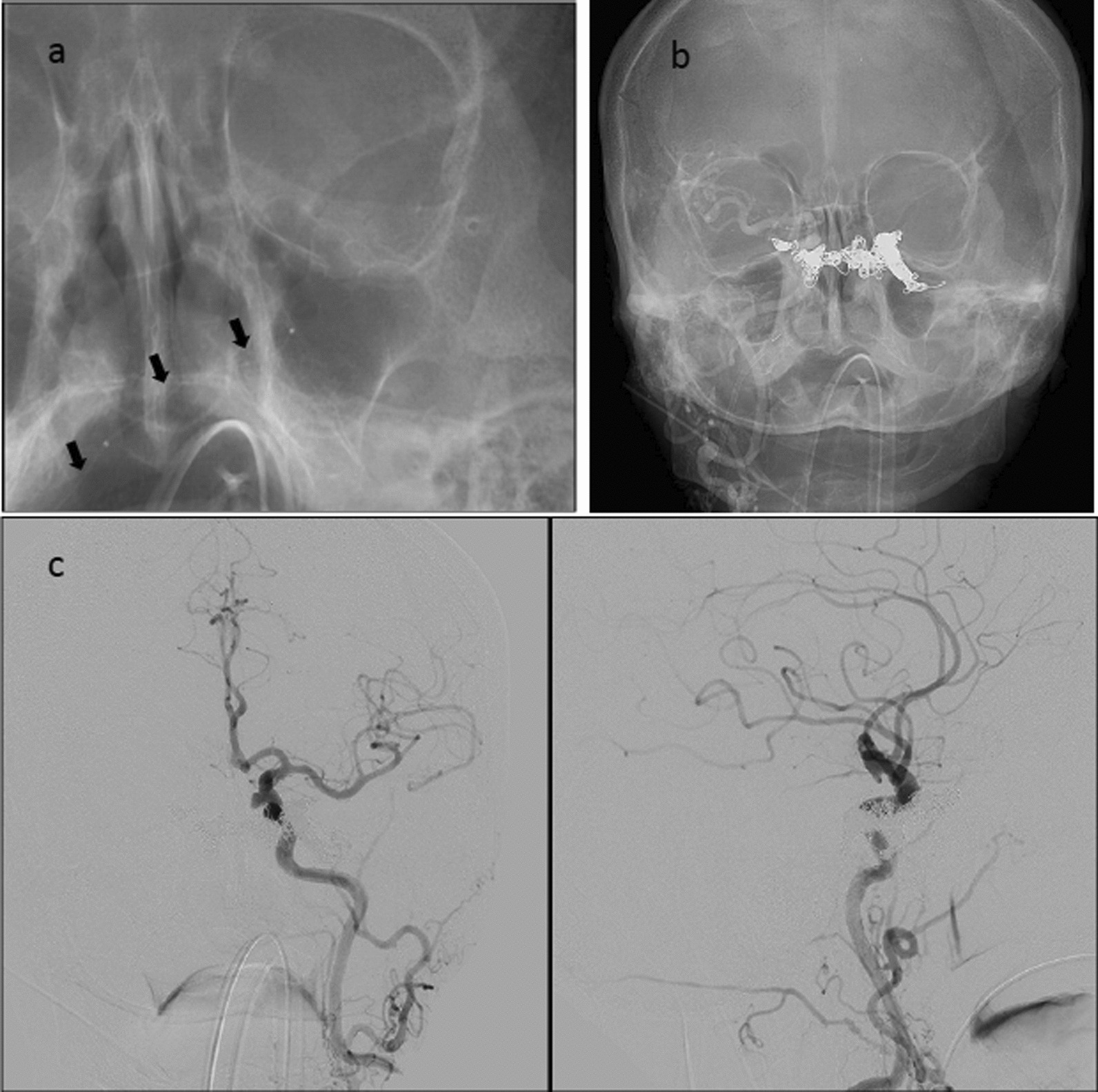
Successful coiling of the indirect carotid-cavernous fistula of case 2. **a** Unsubtracted image shows the 0.017-inch microcatheter (black arrows) passing from the right to left cavernous sinus via the right inferior petrosal sinus. **b** Unsubtracted image shows coil mass spanning the left, intercavernous, and right cavernous sinuses. **c** Angiogram of the left common carotid artery in anterior-posterior (left) and lateral (right) views demonstrates resolution of the carotid-cavernous fistula

Embolization was performed via a right common femoral vein (CFV) access. A 5F guide catheter was positioned in the right IJV. A Headway 17 microcatheter was navigated to the left cavernous sinus fistulous point via the intercavernous sinus (Fig. 2a). Concerto nylon fibered coils (sizes range from 9 to 10 mm) were used to occlude the left cavernous sinus fistulous point. The Headway 17 microcatheter was subsequently navigated to the right cavernous sinus fistulous point, and Concerto fibered coils again were used for occlusion of the fistula (Fig. 2b). Completion angiogram showed complete occlusion of the left CCF and only minimal residual fistula on the right with antegrade drainage into the right IPS (Fig. 2c). No complication occurred during the procedure.

The patient recovered well with resolution of right eye esotropia on 3-month follow-up appointment. No postprocedural imaging was performed, as the patient was lost to follow-up.

## Discussion

Endovascular treatment is the primary treatment for CCFs. While transarterial approach is the preferred method in occluding high-flow direct fistula, there are disadvantages to the approach when it comes to indirect fistula, namely tortuosity of the ECA branches which supply the CCF as well as propensity to lead to cranial nerve palsies [5]. Transvenous embolization has been well-established in the occlusion of indirect CCFs, which reports a 70–90% success rate [6]. IPS is the most common pathway to access the cavernous sinus, which is the route we utilized in our two cases [7].

In our institution, we prefer the use of low profile (0.017-inch) microcatheters to aid navigation through the complex and variable compartments of the cavernous sinus. Occlusion of the CCF is then carried out with coils as demonstrated in the two cases of this series. A downside to using low profile microcatheters is that they are mostly only compatible with bare metal coils. We prefer the use of large diameter fibered coil, as they have superior thrombogenicity to bare metal coils. This allows us to occlude the fistula faster and with less coils deployed. However, most fibered coils require the use of larger microcatheters (0.021 inches and larger). These larger microcatheters are more difficult to track and navigate within the cavernous sinus. Based on the IFU of the Concerto nylon range of coils, those with diameter greater than 4 mm requires a microcatheter with a minimum of 0.021-inch inner diameter [8]. However, we have shown that it is possible to deploy Concerto nylon coils up to 10 mm in diameter with a 0.017-inch microcatheter without technical issue. To our knowledge, the Concerto nylon coils are the only detachable fibered coils in the market which can be deployed via a 0.017-inch microcatheter, especially at its large size.

We have demonstrated feasibility of using a low profile (0.017-inch) microcatheter to deploy larger diameter fibered coils (up to 10 mm) in transvenous embolization of indirect CCF. We hope this report will help other proceduralists in their device selection for transvenous embolization of indirect CCF.

## Conclusion

Our case series highlights the possibility of deploying large diameter (up to 10 mm) Concerto fibered coils through a low profile (0.017-inch) microcatheter in an off-label manner for transvenous embolization of indirect CCF.

## Data Availability

Not applicable.
